# To study the correlation between cognitive decline and Parkinson’s disease with sarcopenia in Qinghai‐Tibet Plateau

**DOI:** 10.1002/alz.087911

**Published:** 2025-01-03

**Authors:** Aiqin Zhu, Jiangang Liu

**Affiliations:** ^1^ Institute of Geriatric, Qinghai Provincial Hospital, Xining China; ^2^ Clinical Research Center for Geriatric Diseases of Qinghai, Xining China; ^3^ Graduate School of Qinghai University, Xining China; ^4^ Qinghai Provincial People’s Hospital, Xining China

## Abstract

**Background:**

To investigate the correlation between cognitive decline and gait speed and grip strength in patients with Parkinson’s disease (PD) and sarcopenia in the hypoxic environment.

**Method:**

From October 2022 to December 2023, a cross‐sectional study was conducted to collect 134 patients (65 males and 69 females) of Parkinson’s disease (age 68.59±10.20 years), who had lived in a Qinghai‐Tibet Plateau area (Qinghai province, an average altitude of 2,500m) for more than 30 years. Skeletal muscle mass index (SMI) was measured using InBody S10 Biospace device from Korea. Grip strength(GS) and six‐meter gait speed test(GST) were measured by JAMAR handgrip dynamometer and Tsinghua Tongfang 6‐meter walking test instrument. PD related scales were used to assess motor and cognitive function.

**Result:**

The prevalence of PD with sarcopenia was 32.8%, including 59.1% (female) and 40.9% (male). Compared with the PD without sarcopenia group, the PD with sarcopenia group showed an increased in age(65.17±8.80 vs 75.50±9.36 years), MDS Unified‐Parkinson Disease Rating Scale‐III (MDS‐UPDRS III) and Hoehn‐Yahr stage (H‐Y) grading (which were used to assess the severity of PD), while Body Mass Index (BMI), GST, SMI, GS, Mini‐mental state Examination (MMSE) and Montreal Cognitive Assessment (MOCA) scores were significantly decreased (P < 0.001). Correlation analysis showed that the GS and GST of PD patients were negatively correlated with UPDRS III (r = ‐0.461; r = ‐0.410), H‐Y grading (r = ‐0.347, r = ‐0.399), and Activities of Daily Living (ADL) (r = ‐0.558, r = ‐0.344) (*P*<0.001), and positively correlated with MMSE (r = 0.452, r = 0.506), MOCA (r = 0.267, r = 0.453) (*P* <0.001), and language ability (r = 0.177, r = 0.208; *P* <0.05). GS was positively correlated with orientation (r = 0.302), attention and calculation (r = 0.262)(P<0.002). Binary regression analysis showed that BMI (OR = 0.682,95%CI: 0.495‐0.940, P<0.05) and MMSE (OR = 0.695,95%CI: 0.557‐0.866, P < 0.001) were protective factors for PD with sarcopenia. H‐Y stage (OR = 4.942,95%CI: 1.567‐15.896, P < 0.006) was a risk factor for PD with sarcopenia.

**Conclusion:**

Cognitive decline is the main risk factor for Parkinson’s disease with sarcopenia in the Qinghai Tibet Plateau region. It may be related to slow gait speed and decreased grip strength. The processing speed and executive dysfunction are mainly related to weak grip strength.

